# Supplementary material for: Cell type transcriptional identities are maintained in cultured ex vivo human brain tissue
Source: bioRxiv. 2024 Dec 20:2024.12.19.629223. Preprint. [Version 1] doi: 10.1101/2024.12.19.629223 (PMC11702615; doi:10.1101/2024.12.19.629223)

bioRxiv preprint doi: https://doi.org/10.1101/2024.12.19.629223; this version posted December 20, 2024. The copyright holder for this preprint (which was not certified by peer review) is the author/funder, who has granted bioRxiv a license to display the preprint in perpetuity. It is made available under aCC-BY 4.0 International license.

Supplemental Figure 1. Marker genes used to assign cell types for tissue A. In cases where multiple marker genes were used, feature sum was used across all the genes listed.

Supplemental Figure 2. Marker genes used to assign cell types for tissue B. In cases where multiple marker genes were used, feature sum was used across all the genes listed.

Supplemental Figure 3. Marker genes used to assign cell types for tissue C. In cases where multiple marker genes were used, feature sum was used across all the genes listed.

Supplemental Figure 4. Marker genes used to assign cell types for tissue D. In cases where multiple marker genes were used, feature sum was used across all the genes listed.

Supplemental Figure 5. Marker genes used to assign cell types for tissue E. In cases where multiple marker genes were used, feature sum was used across all the genes listed.

Supplemental Figure 6. Marker genes used to assign cell types for tissue F. In cases where multiple marker genes were used, feature sum was used across all the genes listed.

Supplemental Figure 7. Gene ontology analysis for all non-neoplastic cell types. The genes showing significant up- or down-regulation in at least two patient samples were compiled and entered into Metascape, a web browser tool that generates gene ontology, cell pathway, and disease features. In none of the cells was a coherent transcriptional shift seen.

Supplemental Figure 8. Gene ontology analysis for all neoplastic cell types. The genes showing significant up- or down-regulation in at least two patient samples were compiled and entered into Metascape, a web browser tool that generates gene ontology, cell pathway, and disease features.

Supplemental Table 1. Up- and down-regulated genes over fourteen days in culture by cell type and tissue in which the genes were identified.

bioRxiv preprint doi: https://doi.org/10.1101/2024.12.19.629223; this version posted December 20, 2024. The copyright holder for this preprint (which was not certified by peer review) is the author/funder, who has granted bioRxiv a license to display the preprint in perpetuity. It is made available under aCC-BY 4.0 International license.

Supplemental Figure 1

6-year-old Dysplastic hemisphere with migrational abnomalities

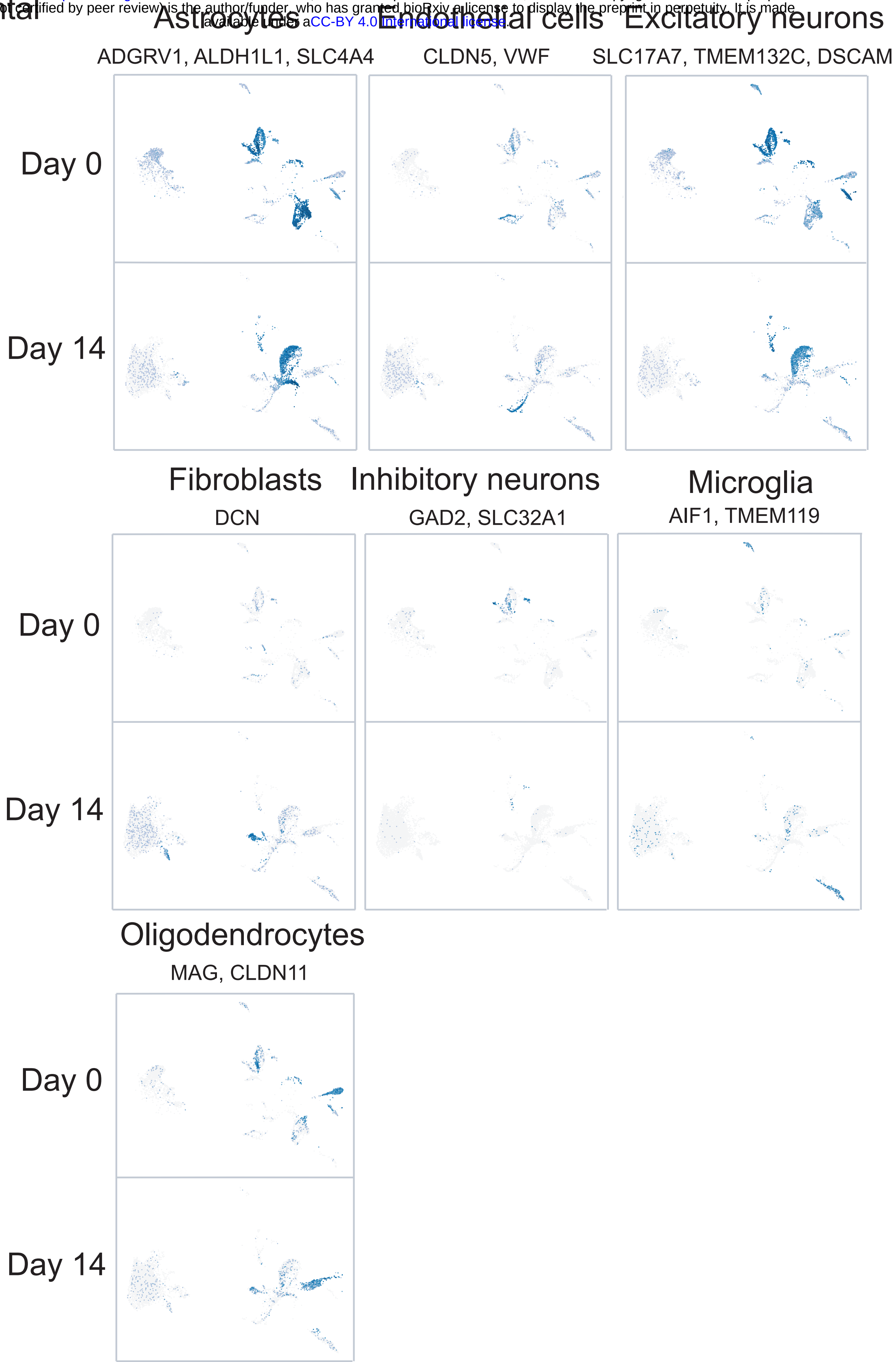

bioRxiv preprint doi: https://doi.org/10.1101/2024.12.19.629223; this version posted December 20, 2024. The copyright holder for this preprint (which was not certified by peer review) is the author/funder, who has granted bioRxiv a license to display the preprint in perpetuity. It is made available under aCC-BY 4.0 International license.

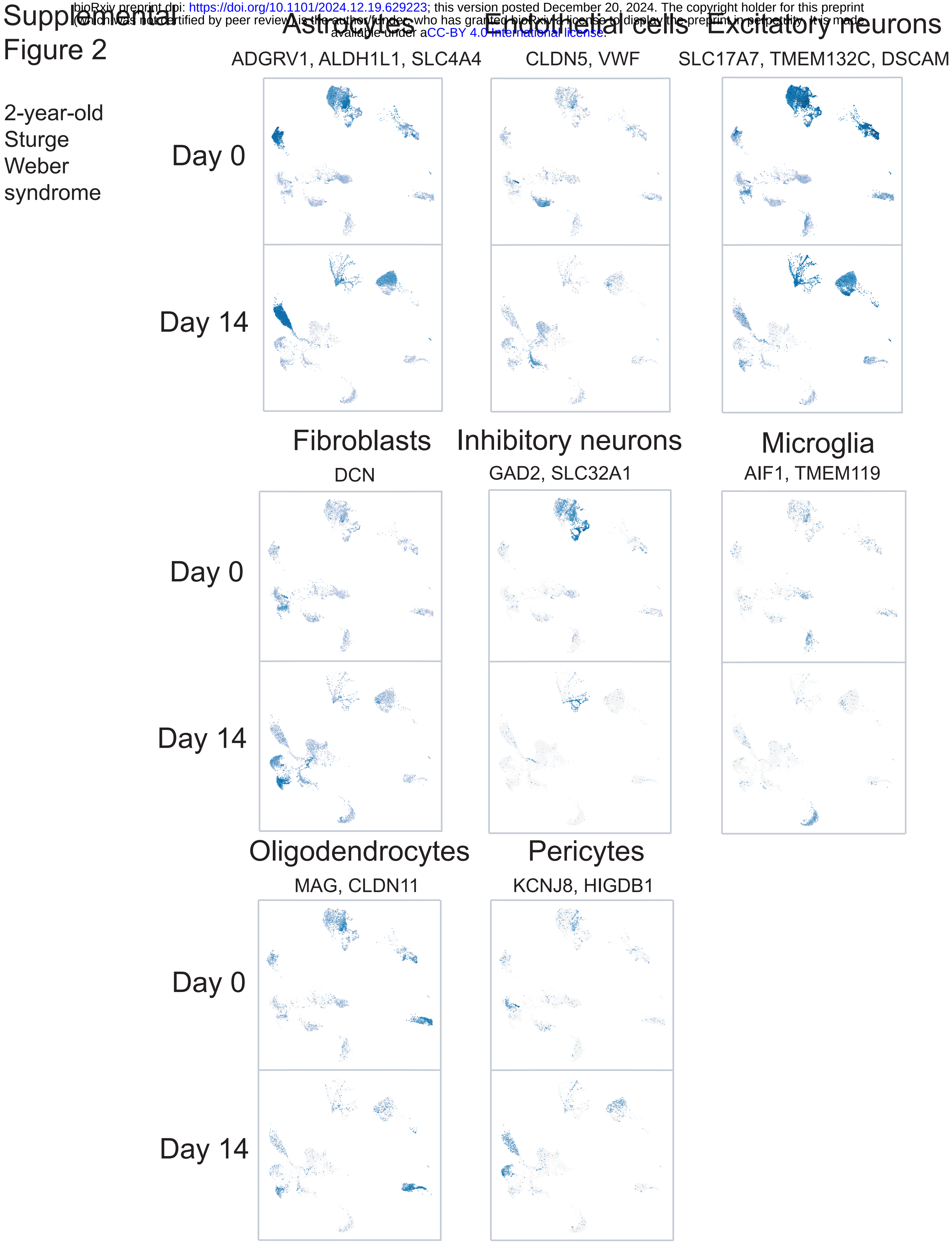

bioRxiv preprint doi: https://doi.org/10.1101/2024.12.19.629223; this version posted December 20, 2024. The copyright holder for this preprint (which was not certified by peer review) is the author/funder, who has granted bioRxiv a license to display the preprint in perpetuity. It is made available under aCC-BY 4.0 International license.

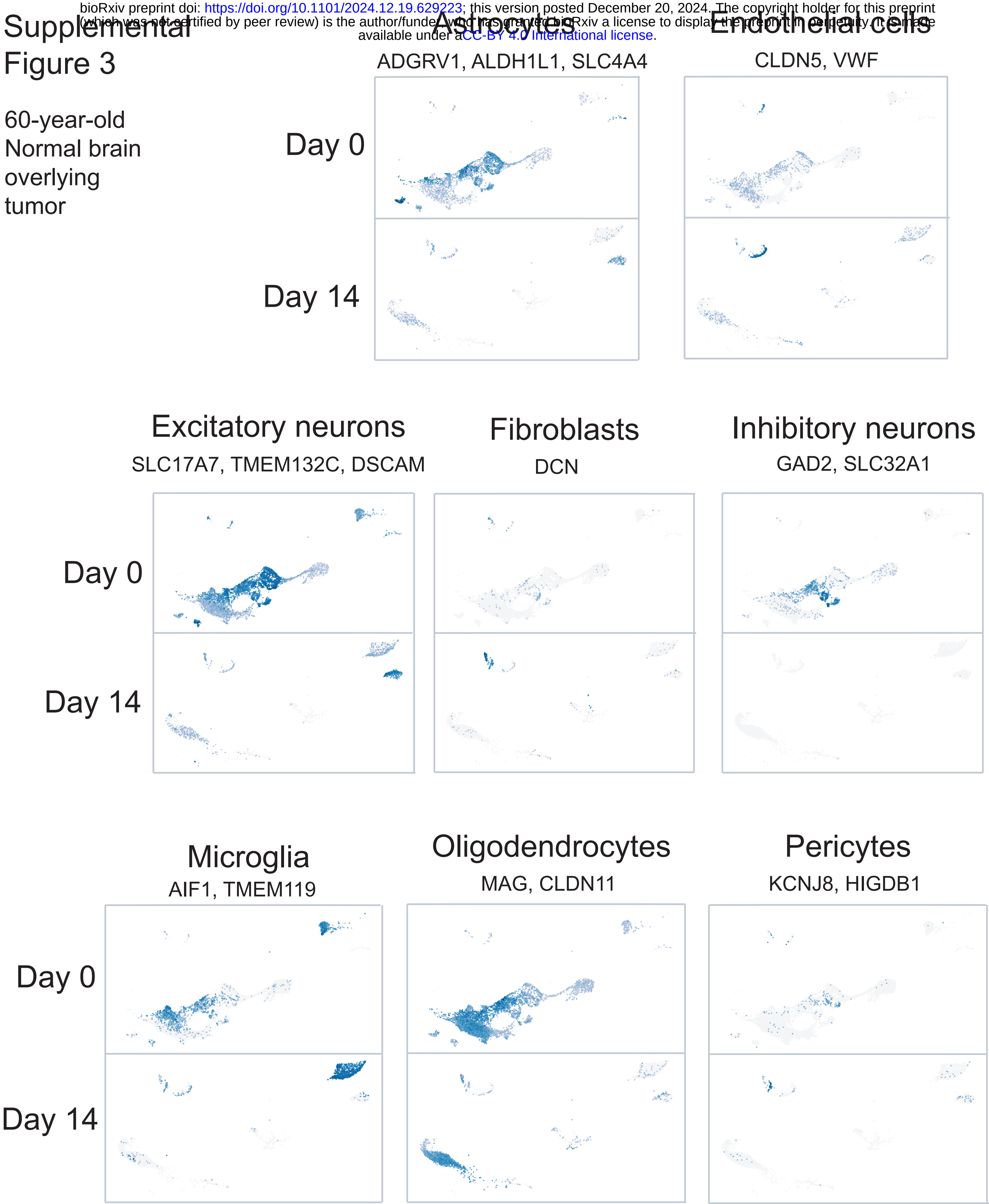

bioRxiv preprint doi: https://doi.org/10.1101/2024.12.19.629223; this version posted December 20, 2024. The copyright holder for this preprint (which was not certified by peer review) is the author/funder, who has granted bioRxiv a license to display the preprint in perpetuity. It is made available under aCC-BY 4.0 International license.

Supplemental Figure 4

86-year-old IDH wild-type glioblastoma

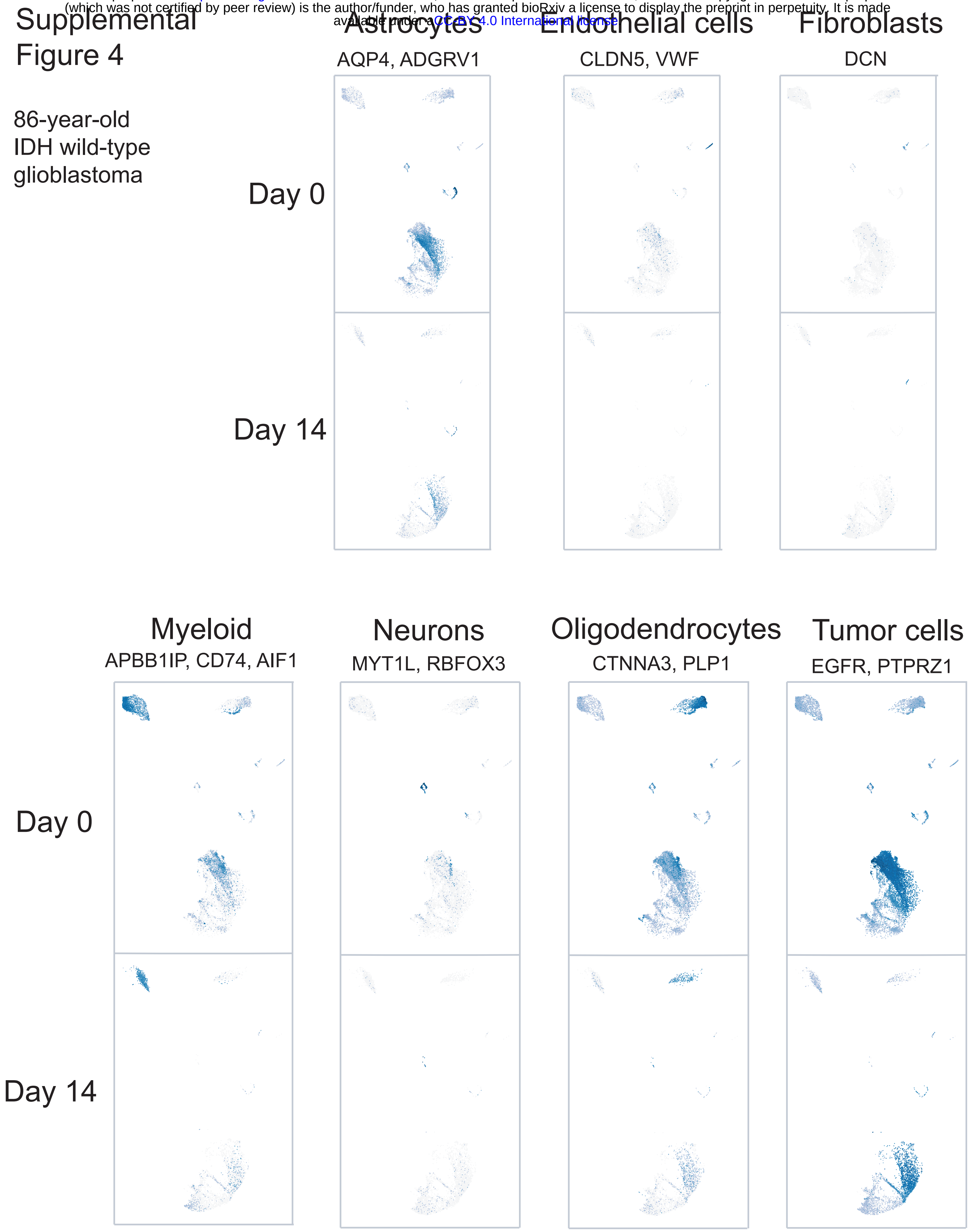

bioRxiv preprint doi: https://doi.org/10.1101/2024.12.19.629223; this version posted December 20, 2024. The copyright holder for this preprint (which was not certified by peer review) is the author/funder, who has granted bioRxiv a license to display the preprint in perpetuity. It is made available under aCC-BY 4.0 International license.

Supplemental Figure 5

65-year-old
Recurrent
IDH wild-type
glioblastoma

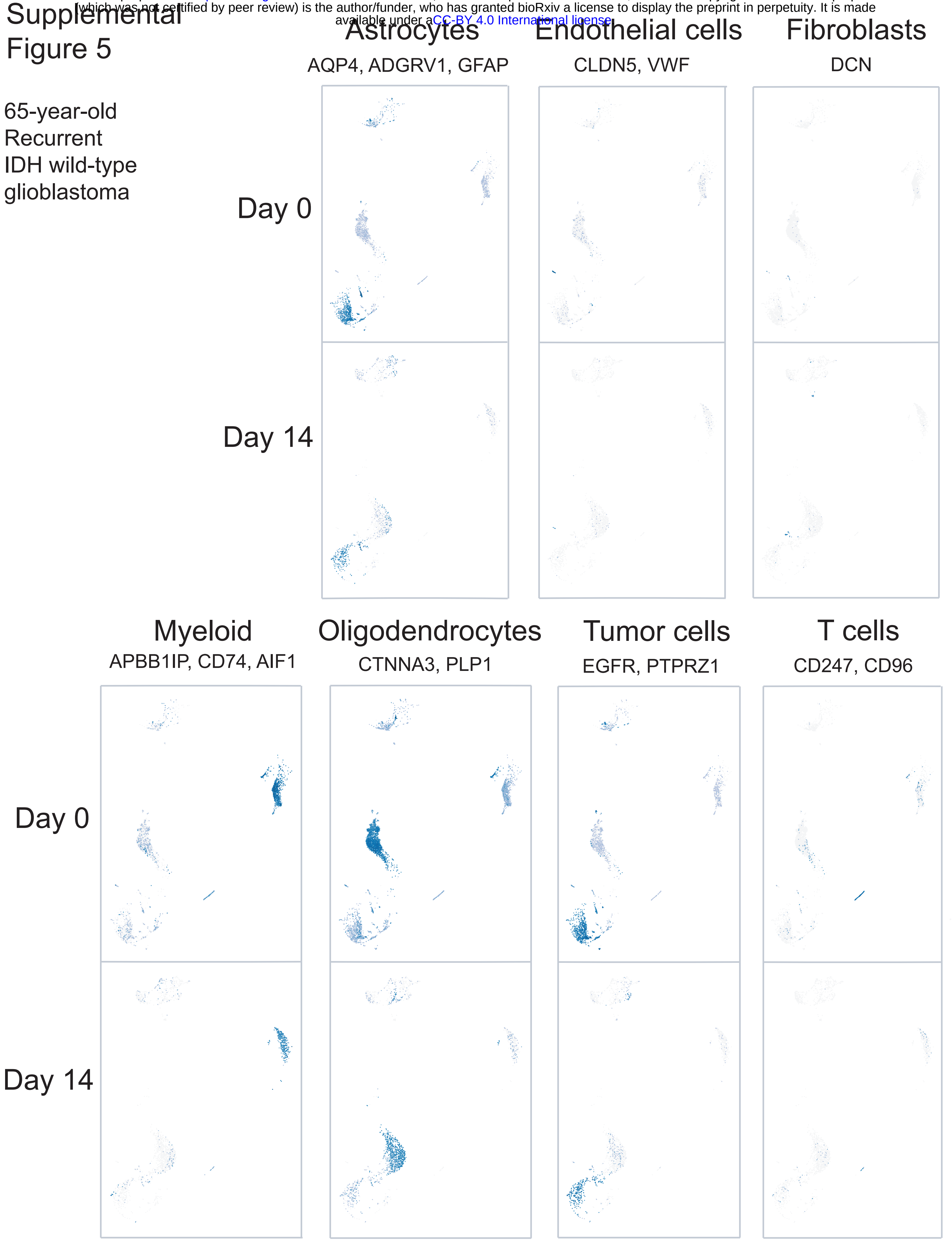

bioRxiv preprint doi: https://doi.org/10.1101/2024.12.19.629223; this version posted December 20, 2024. The copyright holder for this preprint (which was not certified by peer review) is the author/funder, who has granted bioRxiv a license to display the preprint in perpetuity. It is made available under aCC-BY 4.0 International license.

Supplemental Figure 6

20-year-old recurrent SHH-type medulloblastoma

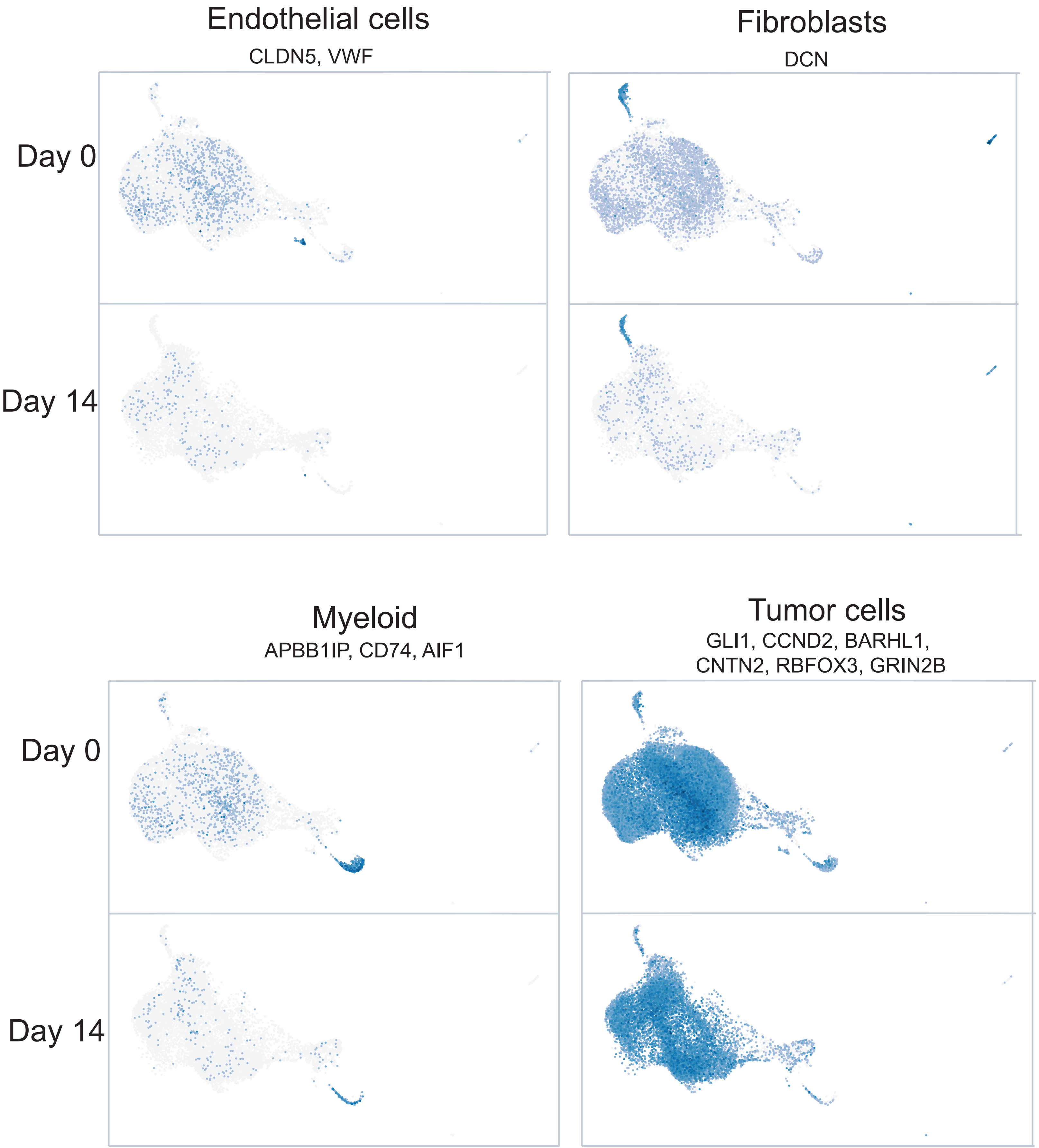

bioRxiv preprint doi: https://doi.org/10.1101/2024.12.19.629223; this version posted December 20, 2024. The copyright holder for this preprint (which was not certified by peer review) is the author/funder, who has granted bioRxiv a license to display the preprint in perpetuity. It is made available under aCC-BY 4.0 International license.

Supplemental Figure 7

Astrocytes up-regulated

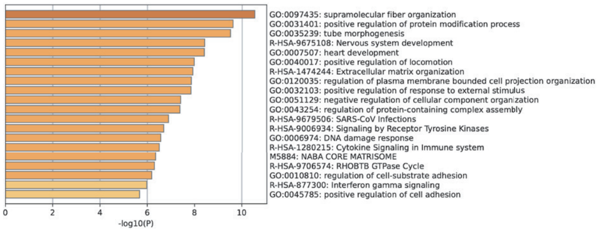

Astrocytes down-regulated

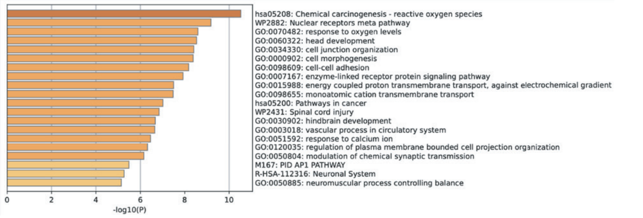

Endothelial cells up-regulated

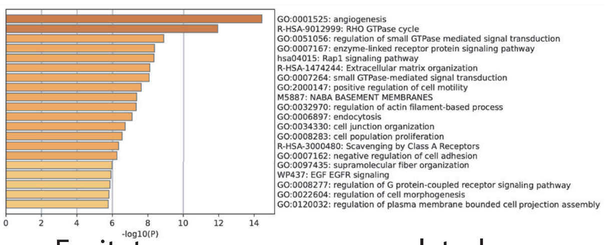

Endothelial cells down-regulated

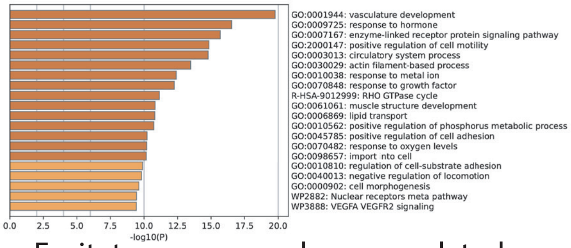

Excitatory neurons up-regulated

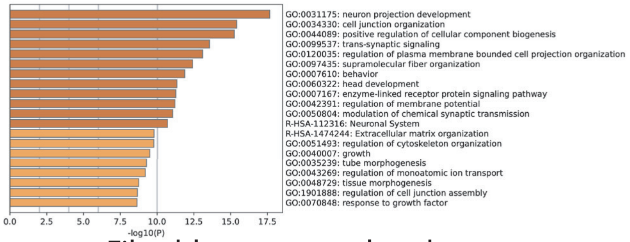

Excitatory neurons down-regulated

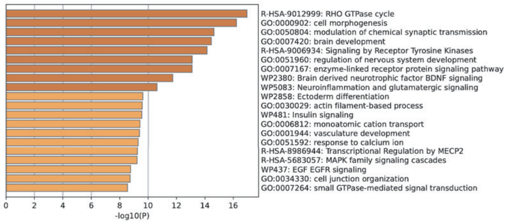

Fibroblasts up-regulated

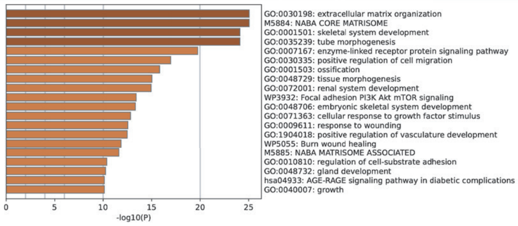

Fibroblasts down-regulated

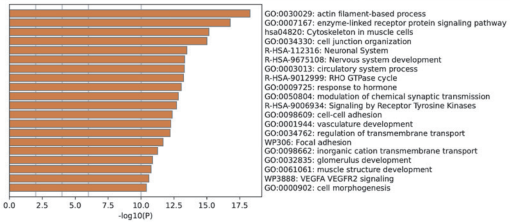

Microglia up-regulated

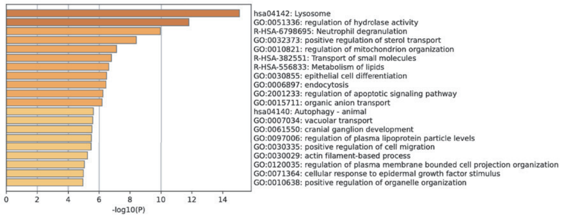

Microglia down-regulated

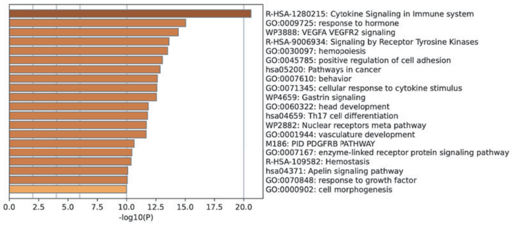

Myeloid up-regulated

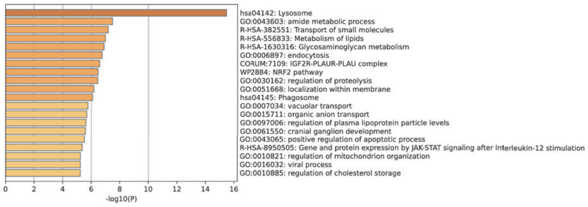

Myeloid down-regulated

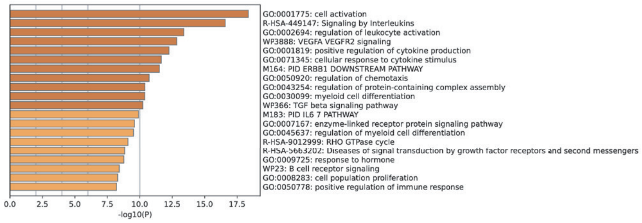

Oligodendrocytes up-regulated

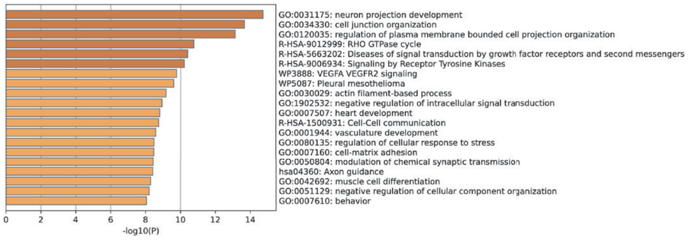

Oligodendrocytes down-regulated

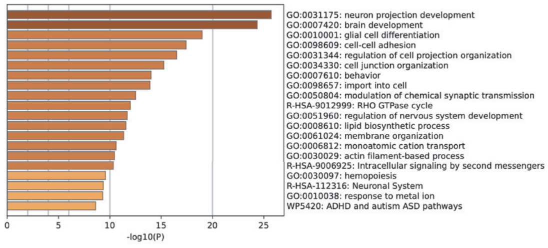

bioRxiv preprint doi: https://doi.org/10.1101/2024.12.19.629223; this version posted December 20, 2024. The copyright holder for this preprint (which was not certified by peer review) is the author/funder, who has granted bioRxiv a license to display the preprint in perpetuity. It is made available under aCC-BY 4.0 International license.

Supplemental Figure 8

Glioblastoma tumor cells up-regulated

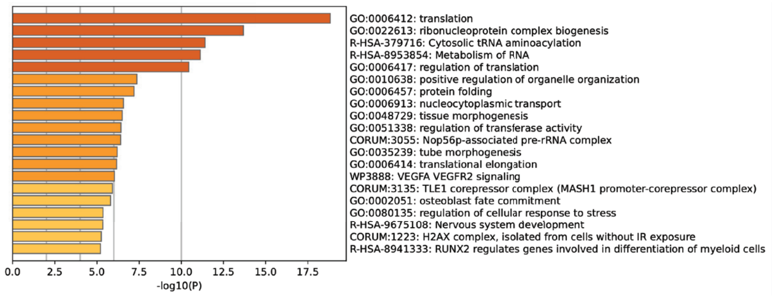

Glioblastoma tumor cells down-regulated

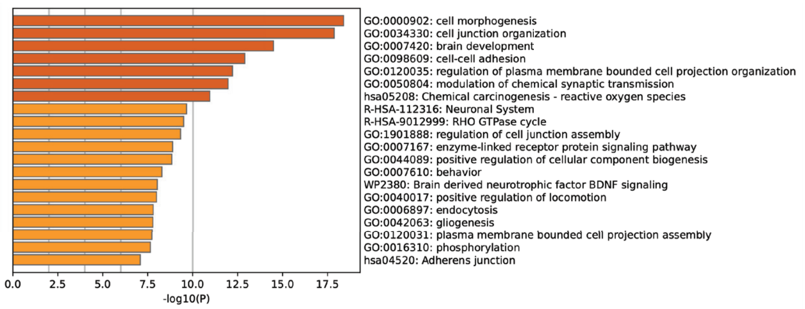

Medulloblastoma tumor cells up-regulated

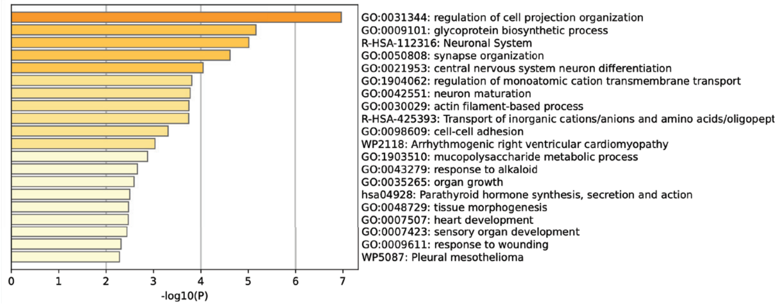

Medulloblastoma tumor cells down-regulated

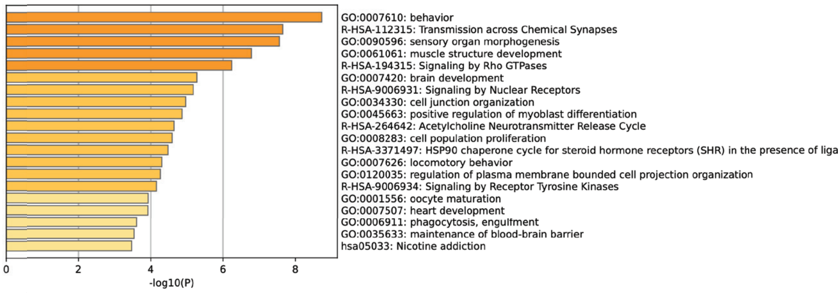

Supplement: Supplement 2 [file NIHPP2024.12.19.629223v1-supplement-2.pdf]
